# Point-of-care HIV early infant diagnosis: is test sensitivity everything?

**DOI:** 10.7448/IAS.18.1.20235

**Published:** 2015-09-04

**Authors:** Lorna Dunning, Nei-yuan Hsiao, Landon Myer

**Affiliations:** 1 Division of Epidemiology & Biostatistics, School of Public Health & Family Medicine, University of Cape Town, Cape Town, South Africa; 2 Division of Medical Virology, University of Cape Town and National Health Laboratory Service, Cape Town, South Africa; 3 Centre for Infectious Diseases Epidemiology & Research, School of Public Health & Family Medicine, University of Cape Town, Cape Town, South Africa

**Keywords:** HIV-exposed infants, EID services, point-of-care, new diagnostic tools, mother-to-child HIV transmission

## Abstract

Despite improvements in PMTCT services in low- and middle-income countries, there are still almost 200,000 new paediatric HIV infections annually in sub-Saharan Africa. This has led to early infant HIV diagnosis (EID) programmes becoming a public health priority, but until recently, EID has required specialist laboratory equipment and trained personnel which is only feasible in urban, centralized facilities. It is thought that the successful implementation of a point-of-care (POC) test for EID has the potential to increase access to virological tests and address some of the barriers regarding retention of infants in care. However, POC evaluation has not integrated focus on performance characteristics with the health systems issues surrounding the adoption of and optimum use of these new technologies. We propose that moderate improvements in linkage to care can more than offset suboptimal sensitivity of a POC EID test which could be critical in adjusting the focus for EID programme management away from test performance and towards their ability to facilitate successful linkage to antiretroviral therapy (ART) services. These findings also highlight the urgent need to explore the implementation and operational aspects of emerging POC tests in order to fully realize the potential benefits of new technologies in practice.

It has been estimated that in sub-Saharan Africa alone almost 200,000 new paediatric HIV infections occur annually during pregnancy, delivery or postpartum via breastfeeding [1,2]. Given the high risk of rapid disease progression in perinatally infected neonates, and research showing that 50% of these infants would die by the age of two if not administered antiretroviral therapy (ART) [3,4], early infant HIV diagnosis (EID) programmes have become a public health priority in many African countries. Indeed, the World Health Organization has recommended early identification of HIV in infants by six weeks of life and immediate referral for initiation of ART for all infected infants under age five to reduce childhood mortality [5]. Yet, despite these recommendations, only about a third of HIV-infected children in low-resource settings receive ART [6].

The process involved in the detection of HIV infection in infants requires nucleic acid amplification tests, performed on specialist laboratory equipment by trained personnel, which are typically limited to urban, centralized facilities [7,8]. In low-resource settings, access to EID is therefore available to a very limited number of children in need, with only roughly 15% of HIV-exposed infants in sub-Saharan Africa receiving an appropriate virological test [9,10]. Meanwhile, there are also major operational concerns around the dried blood spot preparation, long turnaround time and non-retention of infants who are able to receive care [7,8,11,12]. As a result, large numbers of infants are lost to follow-up between HIV testing, the return of results and referral to paediatric ART services [11,13,14]. The successful implementation of a point-of-care (POC) test for EID has the potential to address some of these barriers by providing a more rapid diagnosis, with the possibility of immediate referral of infected infants to appropriate care, resulting in turn in improved child health outcomes [6].

In recent years, there has been significant investment in the development of HIV diagnostic technologies for EID, including a range of POC devices [15,16]. POC diagnostics do not require a sophisticated laboratory setup and can be operated by health care workers without laboratory training [17]. Ongoing field studies have shown that, unlike current gold standard tests, POC tests can provide same-day diagnoses in a reliable, rapid, affordable, simple and robust manner [18].

While there are many EID POC devices in the pipeline, one of the barriers to implementing these products in settings where they are urgently needed is the highly scrutinized evaluation for performance in clinical and field settings [19]. The development and evaluation of POC devices for EID are focused primarily on test performance characteristics, namely sensitivity and specificity compared to laboratory-based gold standards. This focus is in contrast to the a major concern of current EID programmes with the ability to link newly diagnosed patients to care. POC devices could deliver a sustainable option for improving health services; however, the implementation of these products can be a major challenge. Due to the high priority placed on test performance characteristics, markedly less consideration has been given to issues surrounding the adoption of and optimum use of these new technologies, meaning vital research is missing from this area.

To understand the relative contributions of test performance versus linkage to care in affecting the overall impact of EID programmes on referring HIV-infected infants to care, we conducted a thought experiment using data from Hsiao's 2013 retrospective study on the linkage of HIV-infected children to ART [7]. We examined how EID test performance may influence the ART initiation under different assumptions regarding implementation and uptake and compared this to Hsiao's previous analysis which found that only 71% children with a positive HIV PCR result also had a subsequent HIV viral load conducted (a marker in this setting of entry into ART services) and were thus considered successfully linked to care.

The results of our thought experiment are shown in Figure 1. This suggests that a given level of infant ART initiation could be achieved by different combinations of test sensitivity and successful linkage of infected infants. For example, the infant ART initiation rate observed by Hsiao (71%) could be achieved with a test that is only ~72% sensitive but allows 99% successful linkage, or alternatively with a test that is 100% sensitive but that sees 70% of children successfully linked. Indeed, if successful linkage to care could be increased by 10% using a POC device, then the sensitivity of the device could be as low as 88% and still achieve the same levels of ART initiation in infected infants.

**Figure 1:**
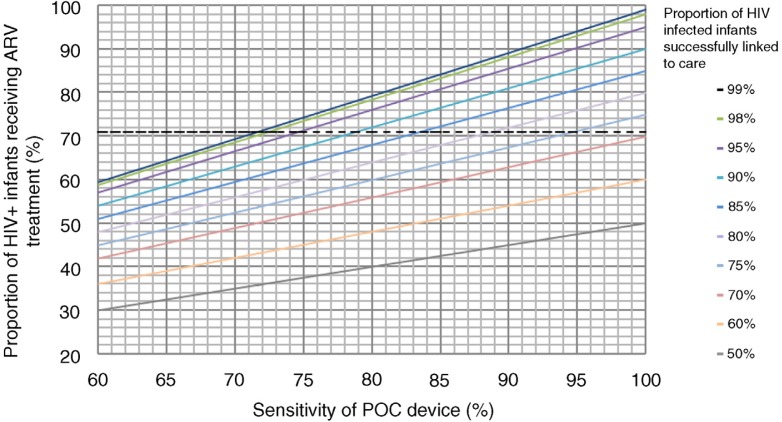
Graph of the proportion of HIV-infected infants who would initiate antiretroviral therapy (y-axis) at varying levels of POC test sensitivity (x-axis) at different levels of successful referral to care based on EID results (ranging from 50–99% based on available literature). The dotted horizontal line at 71% represents the proportion of infants initiating ART as observed previously (7).

POC tests for EID have the potential to improve patient linkage to care and therefore increase the overall proportion of infants who receive ART. This analysis adds insight by suggesting that moderate improvements in linkage to care can more than offset suboptimal sensitivity of a POC EID test. Moreover, for tests with less than perfect sensitivities, repeat testing protocols may assist in maximizing overall performance in some settings.

Whilst it is crucial to carry out rigorous validations on new diagnostic tools before they are released for general use, we wonder if the evaluation of POC tests for EID is not focused somewhat myopically on test performance with much less attention to the ability of such tests to facilitate successful linkage to care within PMTCT programmes.

In order for diagnostic tests to be beneficial and useful to the population for which they are intended, they must be affordable, easy-to-use and provide simple and accurate results that are easily interpreted by health care workers [20]. We argue that POC tests for EID should be judged as much by their ability to facilitate successful linkage to ART services as by traditional test performance characteristics. More generally, there is an urgent need to explore the implementation and operational aspects of emerging POC and near-patient tests in other areas of HIV prevention, care and treatment, in order to fully realize the potential benefits of new technologies in practice.
